# Evaluation of Statistical Process Control Techniques in Monitoring Weekly Body Condition Scores as an Early Warning System for Predicting Subclinical Ketosis in Dry Cows

**DOI:** 10.3390/ani11113224

**Published:** 2021-11-11

**Authors:** Shangru Li, Xiaoli Wei, Jiamei Song, Chengrui Zhang, Yonggen Zhang, Yukun Sun

**Affiliations:** 1 College of Animal Sciences and Technology, Northeast Agriculture University, Harbin 150030, China; lisr1010@163.com (S.L.); songjiamei1120123@163.com (J.S.); Cruivan8254936@163.com (C.Z.); 2 College of Electric and Information, Northeast Agricultural University, Harbin 150030, China; wxllsz@neau.edu.cn

**Keywords:** dry period, body condition score, postpartum diseases, subclinical ketosis, statistical process control

## Abstract

**Simple Summary:**

The management of body condition scores of dairy cows during the dry period affects postpartum health. It is therefore important to monitor the body condition scores so as to predict the occurrence of postpartum diseases. In this study, statistical process control technology was used to monitor body condition scores and to explore its effects on nutritional strategies and the development of postpartum subclinical ketosis. The results showed that the statistical process control technology was not a good tool for the diagnosis of postpartum subclinical ketosis, but an appropriate tool for the guidance of nutritional strategies.

**Abstract:**

The management of body condition score (BCS) during the dry period is associated with the postpartum health outcomes of dairy cows. However, the difference between the actual BCS and the fixed ideal value is not able to accurately predict the occurrence of postpartum diseases. This study aimed to use statistical process control (SPC) technology to monitor the BCS of dry cows, to evaluate the effect of control charts on nutritional strategies, and to explore the utility of SPC in predicting the incidence of postpartum subclinical ketosis (SCK). The BCS and SCK data of 286 cows from the dry off period to 60 days postpartum were collected to set up the early warning function. Three control charts, including a control chart for the average BCS of the herds, for the BCS of each dry cow, and for individual BCS, were established. The early warning signs for postpartum SCK development were: (1) an individual BCS more than 3.5 that remained unchanged for six weeks; (2) a capability index (CPK), an SPC tool, greater than −0.52. Using these parameters, the early warning signs of SCK development were verified in 429 dry cows. The results showed that the accuracy of early warning signal was 0.64 and the precision was 0.26. The control chart showed that the average BCS of dry cows was consistently higher than the expected upper limit of BCS during the experimental period, and that the addition of new cows to the herds increased the average BCS. In summary, the application of SPC technology to monitor the BCS of dry cows was not a good tool for the prediction of postpartum SCK occurrence but was an appropriate tool for guiding positive nutrition strategies.

## 1. Introduction

The dry period, which is the period approximately 60 days before expected calving, presents an opportunity to alter management and feeding strategies for dairy cows to enter their transition period smoothly. Since the energy intake of dairy cows in early lactation is lower than the energy consumed for milk production, negative energy balance (NEB) during this period may lead to an increase in the level of ketone bodies (such as acetone, acetoacetic acid, and β-hydroxybutyric acid (BHBA)) in the blood, leading to the development of subclinical ketosis (SCK) and clinical ketosis (CK) [1,2]. SCK is a metabolic disorder that often occurs during the perinatal period of high-yielding dairy cows [3]. The economic impact of SCK and related diseases is mainly manifested in the reduction of milk production, milk waste, treatment costs, prolonged calving interval and disease elimination costs [4]. At present, the diagnosis of SCK and CK is based on the evaluation of BHBA concentration in blood or milk samples from the cows. A diagnosis of SCK is made when the concentration of BHBA exceeds a certain threshold. However, to achieve the ultimate goal of preventing SCK, early prediction of the risk of SCK is required.

The body condition score (BCS) is a tool that estimates body fat reserve indirectly when monitoring energy intake for dairy cows during the lactation period. The loss of BCS in cows was found to begin 21 d before calving, suggesting that the body condition during the dry period was an indicator for the occurrence of immune problems resulting from an energy deficit [5]. Subsequently, a strong correlation between loss of BCS during the dry period and negative health has reinforced the perception that successful lactation starts during the dry period [6].

An elevated BCS during the dry period is associated with dystocia, metabolic diseases, and poor postpartum udder health [7,8,9]. _ENREF_3Duffield et al. [10] showed that dairy cows with a BCS equal to or greater than 4 during the dry period were associated with a 1.6 times greater incidence of ketosis after calving compared to cows with a low BCS. Findings from a study by Rathbun et al. [9] were consistent with this conclusion. However, the results from the study by Rathbun showed that BCS in the transition period cannot predict the occurrence of ketosis. This conclusion was in contrast to a previous conclusion that a BCS greater than 3.5 during parturition increases the risk of ketosis [11]. The reason behind the difference in the two conclusions was analyzed by Barletta et al. [5] who found that BCS began to change before calving, but this change was not regular. The various forms of BCS changes are complex and inefficient in predicting postpartum diseases. Therefore, new early warning strategies during the dry period should be developed, and nutrition strategies should be adjusted in time to ensure the best BCS, which is associated with the maintenance of optimal immune function and health.

Statistical process control (SPC) technology is a tool for the analysis and evaluation of the production process as well as the timely discovery of abnormal signs of systemic factors based on feedback information [12,13]. It is considered to be a better alternative for monitoring livestock production systems as it provides farmers with an option for monitoring abnormalities and taking measures to eliminate their effects. Mertens et al. [14] used chicken flock data to develop a cumulative sum control chart that alerts managers about important problems in production that have gone unnoticed. Maselyne et al. [15] developed a synergistic control system based on SPC for monitoring the health of fattening pigs. The control chart monitored the registration times and average feeding interval of pigs. Results from the study showed that a problem could be detected within 1.3 days of its inception. An SPC chart was used to monitor the health of calves by generating a control chart for daily average feeding behavior, milk consumption and times of refusal to visit. Although the use of any signal or combined signals has no obvious advantage over observing calves, it provides a better alternative to manual observation [16]. Further studies have indicated that the capability index (CPK), an SPC tool, can replace somatic cell count in monitoring the health of udders in dairy cows, which provides a valuable reference for optimizing SPC [17].

The purpose of this study was to evaluate the use of SPC techniques during the dry period to predict the incidence of SCK in postpartum cows. We hypothesized that the weekly monitoring of BCS using the control chart during the dry period can determine the nutritional needs of dry cows, and that the application of CPK in SPC can predict the incidence of postpartum SCK.

## 2. Materials and Methods

A total of 715 Holstein cows, from Wandashan Dairy Group, Hulan District, Harbin, Heilongjiang Province, China, were included in this study. Mean ± SD parity was 2.56 (±0.44) with a range of 2–4. The cows were housed in freestall barns with sand bedding equipped with self-locking head gates at the feed line during the entire period. Cows at dry off with a mean of 287 d of lactation were transferred to dry groups. Cows at 24 d before the calving date were housed in transitional groups. During the observation period, cows were fed with 3 diets. The first diet was for cows at day 60 to day 25 before the expected calving date and cows that weighed 680 kg with a BCS of 3.3 (National Research Council, NRC, 2001). The second diet was designed to meet the nutritional requirements for the cows in the transition period (NRC, 2001), while the third was formulated to meet the requirements for early lactating cows weighing 650 kg and producing 45 kg of 3.5% fat corrected milk (FCM) (NRC, 2001).

The weekly BCS was monitored manually after morning milking when cows were feeding with the neck clip locked. A scale of 1–5 with 0.25 increments was used to assess BCS based on visual and tactile techniques [18]. The BCS was performed by ultrasonic measurement of backfat thickness (BFT), which was performed by an experienced sonographer using a portable real-time ultrasound (BW-2017, Beishiwo Technology Ltd., Shenzhen, Guangdong Province, China), which corrected the manual score to avoid multiple-score instability. Operation was performed according to Yukun et al. [19]. The dry cows were assessed to determine if the individual cow was too thin (less than 2.75 points), too fat (more than 3.25 points), or in ideal condition (2.75–3.25 points). The disease information was used to validate the alarms produced by the control chart when monitor BCS. The lactating cows as well as the dry cows were assessed according to the order of return from the milking parlor. In addition, cows in the transition period whose BCS exceeded 4 were noted and measures taken to prevent the development of ketosis in early lactation (0~60 days postpartum).

Data for the development of ketosis in the cows were collected from the day of calving to 60 days after calving. The cows were considered to have SCK if the concentration of BHBA was greater than 0.96 mm during routine checks (the concentration of BHBA in blood samples was measured for all dairy cows three days and five days postpartum). Meanwhile, if the cow has one of the following situations:

(1) The veterinarian found that the cow was sluggish during daily inspections.

(2) The smart collar system (Nedap CowControl, Groenlo, Netherlands) issued an early warning of abnormal rumination volume.

(3). Milk production decreased significantly for 2 consecutive days.

A urine ketone test strip (KetoStix, Bayer Diagnostics, New York, NY, USA) was used for testing [20]. If it is “moderate” or above, the cow was considered to have SCK.

### 2.1. Statistical Process Control

The BCS of the dry cows was analyzed every Monday using the control chart. During the monitoring period, cows left the observation group 24 d before the calving date while new cows were successively put under observation. The recommended BCS value for the entire dry period is 2.75–3.25. BCS is considered a stable data set since it has a narrow variation within a short time. For the development of SPC as a management tool, the overall nutritional reserve was assessed and then a correlation model was established between the changes in body condition score and postpartum ketosis. Thereafter, the utility of SPC as a factor for the early prediction of postpartum ketosis was evaluated.

### 2.2. Construction of Warning Functions

In this study, control charts (also known as Shewhart charts) in SPC were used to monitor the management of dry cows. 286 cows were used to set up the early warning function (Table 1). Three control charts were established: one chart monitored the average BCS of the cows in the dry period to determine if the group BCS was within the controllable range according to the historical data; the second chart evaluated the BCS of each dry cow to check if it exceeded the control limit and the average BCS of the group; while the third chart was used to assess the influence of system errors on individual samples from each cow. There were five lines in the control chart: the center line represented the average value of the measurement, while the upper and the lower lines represented the control limits calculated based on the average value and set to ±3 standard error (±3 σ). The other two lines represent the expected level (2.75 to 3.25), used to calculate the process capability index (CPK).

BCS: body condition score It is possible for the cows to deviate from these standard control limits, although it is considered that the cows can be controlled within the upper and lower limits. In theory, there is the possibility of bilateral out-of-range, so the system sets the detected BCS range from 2 to 4. However, unilateral out-of-range is what is normally observed. The average value in the control chart was compared to the expected level (2.75–3.25) in order to adjust or maintain the nutrition strategy. If the individual sample was considered to deviate from the average BCS of the group, the cow was segregated from the rest of the group so as to adopt a nutrition strategy to correct the BCS. The determination of the uncontrolled sample was done by referring to the control chart for individual BCS. The data was labeled as “suspected” data if the value of the uncontrolled sample was greater than 0.5 compared with the last measured value. This was because a substantial change in the observed value may have been due to a systematic error, or the rapid decline in physical condition caused by the disease, and needed to be checked again. The criteria for predicting anomalies using the control chart of dry cows in one barn were: (1) BCS beyond the centerline ±3 σ; (2) a consistent decrease or increase in 6 BCS values in a row.

The control chart is designed to carry out two main functions, namely the separation of cows for individual feeding, and the detection of early warning signals for the occurrence of ketosis. When the BCS of an individual cow exceeded ±3 σ, the cow was separated from the group to adjust its nutrition. Two conditions were set as high-risk early warning signals: (1) when the individual BCS exceeded 3.5 and remained unchanged for 6 weeks; or (2) a CPK value greater than −0.5. This was after taking into account the degree of changes in physical condition, the initial BCS and other factors, while taking CPK as the reference standard. CPK is a parameter commonly used in SPC theory to reflect the level of production management. Its value was calculated using the following formula:

$$\mathrm{CPK}=\mathrm{MIN}\left\{\frac{\left(\mu -T_{L}\right)}{3\sigma },\frac{\left(T_{U}-\mu \right)}{3\sigma }\right\}$$

where μ is the mean value of individual body condition scores during the dry period, and σ is the standard deviation. T_L_ represents expectation offline and T_U_ represents expectation online.

### 2.3. Application of SPC

The performance of SPC was determined using 429 dry cows and was done by comparing the high-risk early warning signals and the actual ketosis cases. The average duration of the dry period in the experimental farm was 42 days. The system sent out high-risk or normal signals after producing 6 BCS data values. If a high-risk signal was generated for a cow and ketosis was diagnosed within 60 days of early lactation, the signal was considered to be a true positive (TP). If the high-risk signal occurred but no ketosis was detected, the signal was regarded as a false positive (FP). If no high-risk warning was issued, and ketosis did not occur within 60 days of lactation, the signal was recorded as a true negative (TN). If there was no warning but ketosis problems occurred, the signal was regarded as a false negative (FN).

## 3. Results

**Figure 1:**
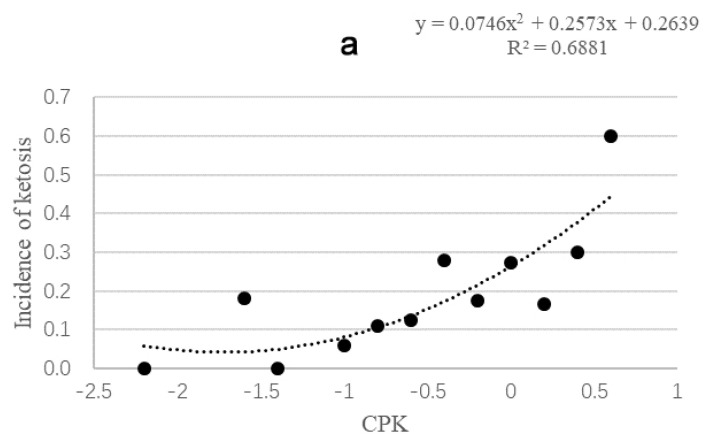

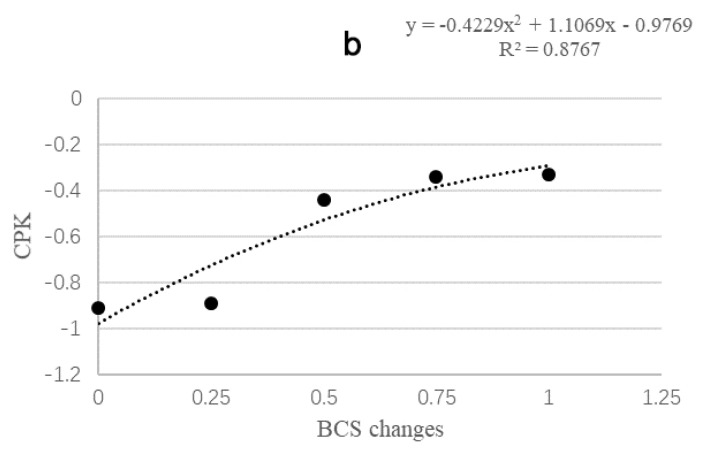
The scatter plot of CPK and BSC changes. (**a**) the model of correlation between capability index (CPK) and the incidence of postpartum ketosis, the risk of ketosis increased with an increase in CPK; (**b**) the model of correlation between CPK and BCS changes, the case where the BCS change is less than 1. When the BCS change is greater than 1, it is considered a system error.

The efficacy of the early warning signals in predicting ketosis was determined using a scatter plot of CPK against the risk of ketosis (Figure 1a). There were 275 dairy cows that met the calculation rules of CPK, and the initial BCS of the remaining dairy cows during the observation period exceeded 3.5, which meant that CPK would not be effective in predicting postpartum ketosis. Therefore, we selected cows whose initial BCS was less than or equal to 3.5 during the dry period for CPK calculation. The risk of ketosis increased with an increase in CPK. When CPK was equal to 0, the risk of cows developing ketosis within 60 days of lactation was 0.26. As seen in Figure 1b, as BCS increased, the value of CPK also increased. When the change in BCS (ΔBCS) was less than 0.25, the CPK was less than −0.72 according to the regression formula, while the risk of ketosis was less than 0.12 according to the regression equation of Figure 1a. On the other hand, when the change in BCS was more than 0.5, the CPK was greater than −0.52, while the incidence of ketosis was more than 0.15. Therefore, the early warning signal for this study was set as a CPK value greater than −0.52.

**Figure 2:**
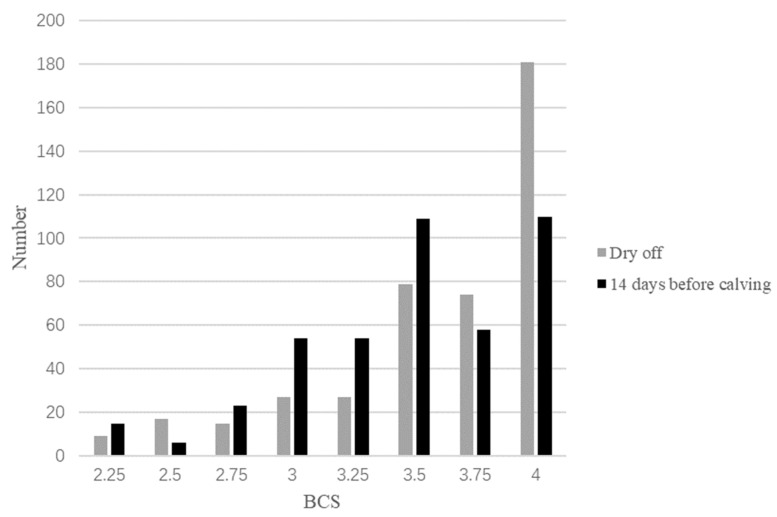
The histograms of cattle participating in SPC functional verification at dry off and 14 days before parturition.

Figure 2 shows the distribution and changing trend of BCS from dry off period to 14 days before calving. The average BCS of the cows at dry off was 3.62, with a BCS of 4 being observed in 0.42 of all cows, and the number of cows with an ideal BCS (2.75 to 3.25) being 69, accounting for 0.16 of all cows. During the dry period, the feed energy density was relatively low, and after adjustment, the average BCS of cows decreased. At 14 days before calving, the number of cows with an average BCS of 3.47 points and greater than 4 points increased by 0.38 points, while the number of cows with an ideal BCS increased by 0.90, accounting for 0.31 of the total number of cows observed. There was little change in the number of thin cows, with the total number of thin cows decreasing from 26 to 21. Although the number of cows with a BCS of 4 points decreased significantly by 0.29, the number of fat cows after adjustment was still high, accounting for 0.65 of all cows. The number of cows with a BCS of 3.5 and 4 points was the highest in the group, 109 and 110, respectively.

The performance of the system is shown in Table 2. A total of 429 dry dairy cows were monitored and a high risk warning was given 174 times. The alarm accuracy was 0.64 ((TP + TN)/N) while precision was 0.26 (TP/(TP + FP)). There were 85 alarms issued due to CPK, with 26 being true positive, at an accuracy of 0.30. There were 89 alarms issued to consistent high levels of BCS, 18 of which actually occurred, with an accuracy of 0.20. The change in BCS of cows with ketosis during the dry period was 0.1 times more than that of healthy dairy cows, and the number of cases of ketosis without alarms was 23.

**Figure 3:**
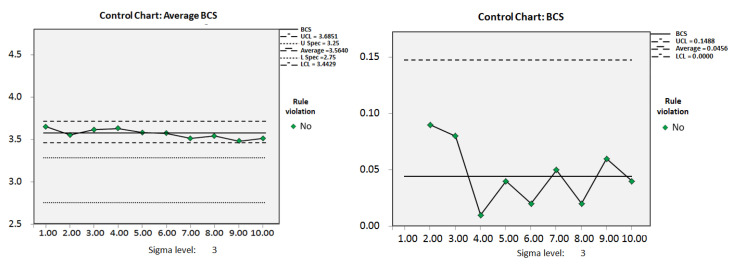
Individual moving range control chart for monitoring average BCS of the herds. **Left**: average BCS; **Right**: BCS changes (ΔBCS). BCS: body condition score; UCL: upper control limit; U Spec: upper specification; L Spec: lower specification; LCL: lower control limit.

**Figure 4:**
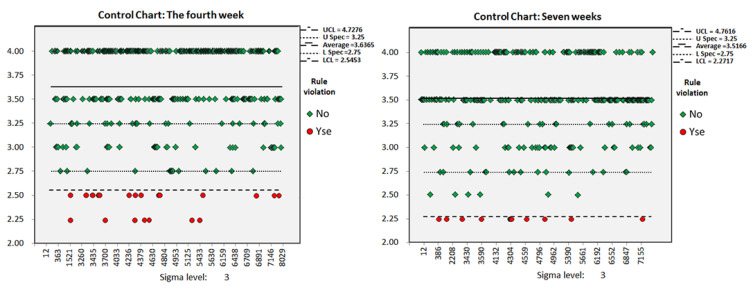
Example of the individual control chart for each BCS of all dry cows in the fourth (**left**) and seventh (**right**) week. The red (alarm) points represent out-of-control observations.

N: number; ΔBCS: BCS changes; SD: standard deviation; BCS: body condition score; CPK: capability index; TP: true positive; FP: false positive; TN: true negative; FN: false negative Figure 3 shows the average BCS of the cows in the dry period during the experiment. The average BCS for the group of dry cows was higher than the ideal BCS at 3.56. The change in BCS was controlled throughout the experiment although the average BCS did not reach the ideal BCS. From the second week to the fourth week, the average BCS of dry-off cows increased from 3.54 to 3.64, and the total number of dry cows increased by 161. From the fourth week to the ninth week, the number of dry cows increased by an average of 38.6 per week, and the average BCS for the group decreased to 3.48 at the ninth week. Figure 4 shows an example of the control chart for the dry cows for the fourth and seventh weeks, which described the BCS for each dry cow. In the fourth week, the average BCS was 3.64, and the lower limit of control was 2.54, indicating that the pasture administrator needed to reduce the feed energy density. Cows with a BCS lower than 2.54 needed to be fed separately, by increasing the feed energy density to ensure maintenance of energy reserves. In the seventh week, the average BCS decreased to 3.52, and the lower limit of control was adjusted to 2.27. Contrary to the fourth week, cows with only 2.5 points were added to the group with the high energy density feed.

## 4. Discussion

For the first time, SPC was used to monitor the BCS in this study. SPC monitored the trend of the changing feeding levels during the dry period. The system was designed to give warning of cows at a high risk of postpartum ketosis and for assessing their energy reserves during the dry period rather than being a tool for the diagnosis of ketosis. Our results showed that, according to the treatment records of clinical diagnosis, the signal of a decline in BCS during the dry period provides the most positive information. In past studies, SPC has been tried as an effective method of monitoring, controlling, and improving processes in livestock production. For example, Quimby et al. [21] controlled the feeding time by applying a self-starting cumulative sum (CUSUM) control chart. Quimby [21] reported that they could detect sick calves 4.5 days earlier than experienced managers. Madsen and Kristensen et al. [22] applied a similar method to pen-level water intake for piglets and found that they could predict diarrhea outbreaks one day before clinical symptoms were observed. In one study, SPC was used to monitor milk yield in order to predict health problems in advance so as to observe the effectiveness of the management strategies [23]. However, the dry period is a critical period that is easily ignored by managers, since the dairy cows do not have obvious economic benefits during this period. In addition, a BCS below 3.5 is a common goal for dairy cows in the dry period, but this fixed expectation does not take into account the variability of the biological systems.

### 4.1. Predicting Health Performance

Body condition score is not a change-sensitive indicator but a reflection of feeding strategies, which can have secondary effects on health and production. Using the changes of CPK in SPC, results from our study suggested a linear relationship between CPK and prevalence of SCK. This finding was consistent with results from other studies and indicated that a change in BCS during the dry period is a positive signal for predicting diseases. Chebel et al. [6] classified dry cows body condition loss as excessive loss (ΔBCS ≤ −0.75), moderately lost cattle (ΔBCS = −0.5~−0.25), constant cattle (ΔBCS = 0) and acquired cattle (ΔBCS ≥ 0.25), and recorded morbidity and production performance at 305 days postpartum. The loss of body fat during the dry period was associated with a higher incidence of uterine diseases and metabolic diseases. In this study, a decrease in BCS in dry cows was associated with health disorders. Findings from a previous study we conducted found that cows with low BCS between 21 days before parturition and calving, secreted more hormones, and a slight decrease in BCS was a warning of postpartum health [24].

Our study did not compare the advantages of managers and SPCs in diagnosing postpartum ketosis, but our system settings provided early warning information, because poor BCS changes during the dry period are early signs of postpartum ketosis. The dry period (excluding the pre-transition period) is about 42 days. In order to ensure a sufficient normal distribution of the observed values, a period length of seven days can be selected as the period of the product graph. This choice is similar to the research of Vries [25]. For monitoring the estrus detection rate, the cycle length of 100 cows is 30 days, and for 1000 cows the cycle length of seven days is a reasonable choice to obtain the first detection signal of short-term out of control.

In our study, we set a fixed expected BCS during the dry period according to the traditionally suggested value. We used this setting because changes in BCS are usually small during a short period, although this setting does not represent characteristic changes in biological systems. In our study, the fat cows at dry off accounted for 0.78 of all cows, but the incidence of ketosis was only 0.16. However, BCS is limited in the prediction of postpartum health based on traditional recommendations. Therefore, there is a need for further research to explore the changes in expected BCS during the dry period so as to accurately predict postpartum performance and health.

### 4.2. Control Chart Function and CPK

One of the aims of this study was to develop an early warning application using automatic BCS detection. In our previous research [19], the accuracy of automatic image BCS model was 0.43, but the accuracy and stability of the system was limited, thus affecting the application of CPK. However, a change in 0.5 units in the BCS gave a CPK value of −0.52. This implied that if the system error was within 0.5 units, the CPK value would be less than −0.52, and there would be no alarm. The accuracy of our image model was 0.9 when the error range of ΔBCS was 0.5, and there was only 0.1 probability that a false alarm caused by an error in the system accuracy would occur. Therefore, it is feasible to use the automatic image BCS equipment for early warning in theory. An additional aim was to determine how to avoid systematic errors.

**Figure 5:**
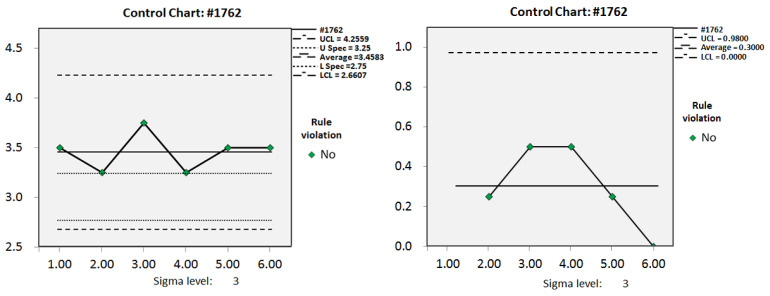
Control chart of BCS of cow 1762. **Left**: Course of individual BCS for 6 weeks. **Right**: BCS changes (ΔBCS).

An important advantage of using CPK over directly using BCS was that it created significant early warning limits: the early warning line of CPK was −0.52. A similar effect of CPK has been reported in previous studies [17]. A logistic regression model between somatic cell count and the prevalence of mastitis can be used to estimate the incidence of subclinical mastitis. It has been suggested that a critical value of 0.26 for subclinical mastitis can effectively prevent the occurrence of subclinical mastitis. The limitation of directly observing BCS was analyzing the variation, so it was more intuitive to introduce CPK value as a reference index in statistics. Additionally, CPK is statistically determined according to the ideal upper and lower limits and population average. Our results showed that higher CPK values due to loss of BCS in dry cows were close to the ideal BCS at dry off. This meant that the prevalence of ketosis was not the same in all dry cows with decreased BCS. This suggests that there is a need to reduce the body fat reserves of overconditioned cows to avoid nutritional metabolic disorders caused by obesity. However, dramatic changes in BCS (ΔBCS > 0.5), or the loss of BCS in thinner cows indicates that nutritional metabolic disorders occurred during the dry period, a finding that has been demonstrated in our previous studies [24]. Another factor that led to high CPK values was that the individual ΔBCS values were the same during the dry off and the transition periods, but changes in BCS that occurred during the intermediate process were reflected in the CPK values (Figure 5). Systematic errors and dynamic nutritional changes of dairy cows can cause CPK fluctuations over a dry period. One of the important reasons for the introduction of CPK was to eliminate the interference of these factors. Whether the BCS of dry cows changed or remained the same, a CPK value less than −0.5 could not be classified as a high-risk factor for the development of disease.

According to Figure 1, CPK was −1.72 when the ketosis caused by a decline in BCS was equal to 0, and a ketosis of 0 was as a result of an initial BCS of 4.15 or 1.58. However, being too fat and too thin in actual production can cause the occurrence of various postpartum diseases, and it is not enough to predict the incidence of disease using CPK alone as an early warning signal. Another method was to monitor cows whose BCS were too high and remained the same during the dry period. Our results showed that this factor caused a 0.22 risk of ketosis, which is a high-risk warning signal. We set a BCS above 3.5 points as an early warning signal based on recommendations by Roche et al. [8]. It appears that it is overconditioning and not low BCS that predisposes cows to an increased risk of periparturient metabolic disorders, since there are several reports that cows above 3.5 have twice the risk of ketosis as cows below 3.5 [11].

The control chart tracks the average BCS and provides suggestions on nutrition strategies according to the relationship between the average value and the expected value. From our study, the average BCS during this experimental period was consistently higher than the expected value. Although the BCS of fat cow was more likely to decline, the dry cows with a BCS lower than 3.25 had a higher risk of ketosis based on the changes in CPK index. Therefore, it was suggested that dry cows should be grouped based on the BCS during the dry period. This was similar to findings by Contreras, Ryan, and Overton [26] that the thinner cows fed with common diet produced the most milk, while fatter cows fed with high energy diet produced more milk than when fed with common diet. On the other hand, the addition of the new herds was seen to increase the average BCS of the herds, which could explain the persistently high BCS in the dry group. We recommend a reduction in the energy levels during the later period of lactation to ensure a lower BCS at dry off. This is likely to improve postpartum performance and the maintenance of cow health.

In summary, two factors with high CPK values were associated with an increased risk of postpartum ketosis in our study. The factors are: (1) a decline in BCS during the dry period in cows below 3.25 at dry off, and (2) a decrease in body condition by more than 0.5, or more than 3.5 at calving. Therefore, monitoring CPK values and body condition scores of cows can predict the deterioration of cow health.

## 5. Conclusions

To evaluate the BCS of dry cows in this study, SPC technology was not an appropriate tool for the diagnosis of postpartum disease, but rather a strategy for developing a positive nutrition guidance program. CPK, which includes BCS classification and changes, is a more appropriate tool compared to using BCS directly in making decisions concerning nutritional strategies of dairy cows during the dry period. High BCS and CPK values are potential predictors of postpartum ketosis, and the control chart can show the gap between the average BCS and the ideal BCS of dry cows for the timely alteration of nutritional strategies.

## Figures and Tables

**Table 1:** Distribution of the number of different BCS for the construction of warning function.

BCS	Dry Off	Transition
2.25	6	11
2.5	11	6
2.75	3	12
3	26	21
3.25	25	22
3.5	45	83
3.75	58	44
4	112	87

**Table 2:** Performance of the early warning for the validation period.

Item	N	ΔBCS	SD
Total	429	−0.09	0.35
Alerts	174	−0.21	0.31
Ketosis	69	−0.17	0.26
Normal	360	−0.07	0.22
Alerts from high BCS	89	0.00	0.00
Ketosis with high BCS	18	0.00	0.00
Alerts from CPK	85	−0.40	0.31
Ketosis with CPK	26	−0.36	0.26
TP	46	−0.22	0.28
FP	128	−0.21	0.24
TN	230	0.01	0.32
FN	23	−0.09	0.25

## Data Availability

All listed data are available.
